# Clinical outcome of the Gant-Miwa-Thiersch procedure for colonic mucosal prolapse after intersphincteric resection—a single-center report from Japan

**DOI:** 10.1016/j.amsu.2021.103005

**Published:** 2021-11-05

**Authors:** Toshikatsu Nitta, Masatsugu Ishii, Jun Kataoka, Sedakatsu Senpuku, Yasuhiko Ueda, Ryo Iida, Ayumi Matsutani, Takashi Ishibashi

**Affiliations:** Division of Surgery Gastroenterological Center Medico Shunju Shiroyama Hospital, Osaka, Japan

**Keywords:** Gant-Miwa-Thiersch procedure, Intersphincteric resection, Mucosal prolapse

## Abstract

**Introduction:**

Dysfunctions such as mucosal prolapse occur after intersphincteric resection (ISR) to treat lower rectal cancer, even when it is possible to preserve the anus.

**Method:**

We analyzed the data of 12 patients with rectal or colonic prolapse who underwent the Gant-Miwa-Thiersch procedure between March 2017 and May 2021.

**Result:**

There were no severe postoperative complications or recurrences.

**Case presentation:**

A 75-year-old Japanese man initially underwent ISR and had mucosal prolapse nine months after his initial operation. We performed the Gant-Miwa-Thiersch procedure for colonic mucosal prolapse after ISR.

**Surgical procedure:**

Our procedure is a perineal plication method of prolapsed colonic mucosa with nylon wiring (The Gant-Miwa procedure), using a 1-nylon wire encircled three times to straighten the anal canal, with a cord inserted above the internal sphincter muscle (Thiersch procedure).

**Discussion:**

Mucosal plication is performed via the Gant-Mowa or Delorme procedure to reduce the risk of recurrence. However, mucosal plication can be performed many times. Our Thiersch procedure involves encircling and straightening the anal canal with a 1-nylon wire to fix the new internal anal sphincter. In conclusion, the Gant-Miwa-Thiersch procedure for rectal and colonic mucosal prolapse, especially after ISR, is a viable treatment option.

## Highlights

• Our procedure is The Gant-Miwa procedure, using a 1-nylon wire encircled three times (Thiersch procedure).
• Mucosal plication is performed via the Gant-Mowa or Delorme procedure to reduce the risk of recurrence.
• The Gant-Miwa-Thiersch procedure for rectal and colonic mucosal prolapse, especially after ISR, is a viable treatment option.

## 1 Introduction

Colorectal cancer is the third most common cancer, and the prevalence of lower rectal cancer has increased worldwide [1]. Intersphincteric resection (ISR) for lower rectal cancer prevents permanent stoma. The operation for lower rectal cancer, including sphincter-saving rectal resection with coloanal anastomosis, is an oncologically accepted procedure because of its low local recurrence rate [2]. ISR, including transanal total mesorectal excision (TaTME) is the ultimate anus-preserving operation [3,4].

However, some dysfunctions, such as increased stool frequency, urgency, clustering, and incontinence, occasionally occur after ISR, even when it is possible to preserve the anus of the patient with lower rectal cancer [[5], [6], [7]]. These dysfunctions are serious problems despite the low postoperative recurrence rate for rectal cancer [7]. Furthermore, ISR is a relatively novel technique, so there is a risk of unexpected and uncommon complications associated with ISR. Uncommon complications include mucosal prolapse and anastomosis strictures [8].

Here, we introduce our classical surgical technique, the Gant-Miwa-Thiersch procedure for mucosal prolapse, especially after ISR, and demonstrate the procedure's performance outcome at our institution.

## 2 Method and outcome of the Gant-Miwa-Thiersch procedure performed at our institution

Twelve patients with rectal or colonic prolapse underwent the Gant-Miwa-Thiersch procedure between March 2017 and May 2021 at our institution, Medico Shujyu Shiroyama Hospital, Osaka, Japan. We analyzed data regarding the age, sex, body mass index (BMI), operating time, operative blood loss, nodules in the sutured mucosa, encircling wire, complications, postoperative interval times, and the clinical course (Table 1, Table 2). The ethics review board of our hospital approved our retrospective review.

**Table 1:** Characteristics of 12 patients undergoing the Gant-Miwa-Thiersch procedure.

	
Age: year	86.3 + SD6.9 years
Male: Female	3 : 9
Body Mass Index	20.6
Operation time	64.5 + SD19 minutes
Bleeding in operation	6.7ml
Nodules in the sutured mucosa	43 + SD25 times
Encircling wire	All 1-nylon 3 ± 1 times
Complication	none
Interval time after operation	29 + SD20 months
**Clinical course**	recurrence: none

**Table 2:** Overview of characteristics of 12 patients undergoing the Gant-Miwa-Thiersch procedure.

	Age (years)	Sex	Body Mass Index	Operation time (minutes)	Bleeding in operation (ml)	Nodules in the sutured mucosa	Encircling wire	Encircling wire times	Complication	Interval time after operation
1	75	male	20	82	5	25	Nylon	3	none	3 months
2	82	female	31.2	48	5	56	Nylon	3	none	6 months
3	74	male	19.7	59	25	25	Nylon	2	none	22 months
4	91	female	18.6	50	5	70	Nylon	4	none	25 months
5	94	female	26.2	45	5	30	Nylon	3	none	27 months
6	90	female	19.7	35	5	29	Nylon	3	none	28 months
7	95	male	18.7	95	5	107	Nylon	3	none	35 months
8	84	female	15.5	90	5	35	Nylon	3	none	51 months
9	89	female	16.9	55	5	27	Nylon	3	none	52 months
10	91	female	22.0	70	5	53	Nylon	3	none	54 months
11	83	female	20.1	80	5	25	Nylon	3	none	69 months
12	88	female	18.0	65	5	38	Nylon	3	none	12 months

## 3 Result of our procedure (Table 1, Table 2)

The sample of 12 patients reviewed included 9 females (75%) and 3 males (25%) with a median age of 86.3 years (range 74–95 years). All patients underwent the Gant-Miwa-Thiersch procedure under general anesthesia. The median operation time was 64.5 minutes (range 35–95 minutes), and blood loss was 6.7 ml. During the Gant- Miwa procedure, there was a median of 43 times (range 25–107 times) that the nodules in the sutured mucosa were left unreduced due to mucosal prolapse. As a prosthesis, we used a 1-nylon encircling wire in the Thiersch procedure. The wire was encircled 3 ± 1 times, and most mucosae were encircled three times. The median interval time after our procedure was 29 months (range 1–66 months), and no severe postoperative complications or deaths occurred. There were no signs of recurrence in any of the 12 patients. Of the 12 cases undergoing ISR for rectal cancer, mucosal prolapse as a unique complication of ISR occurred in only one case (1/12: 9%).

## 4 Case presentation of mucosal prolapse after intersphincteric resection

**Fig. 1:**
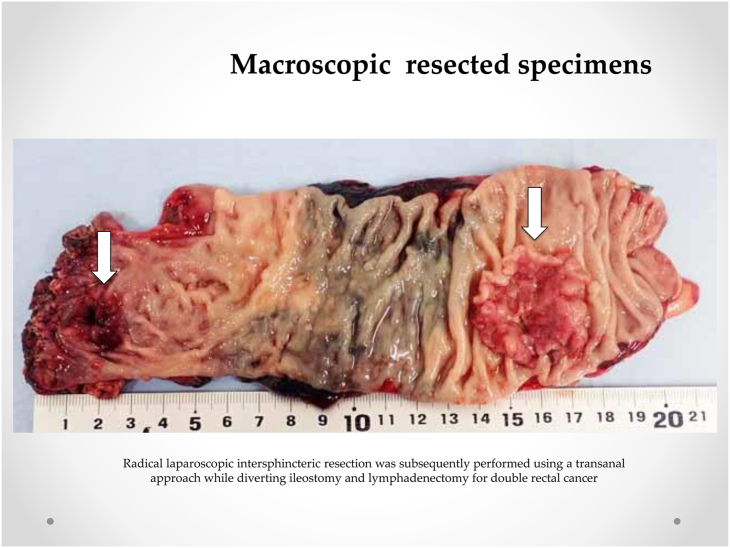
**Macroscopic resected specimens.** Radical laparoscopic intersphincteric resection was subsequently performed using a transanal = approach while diverting ileostomy and lymphadenectomy for double rectal cancer.

**Fig. 2:**
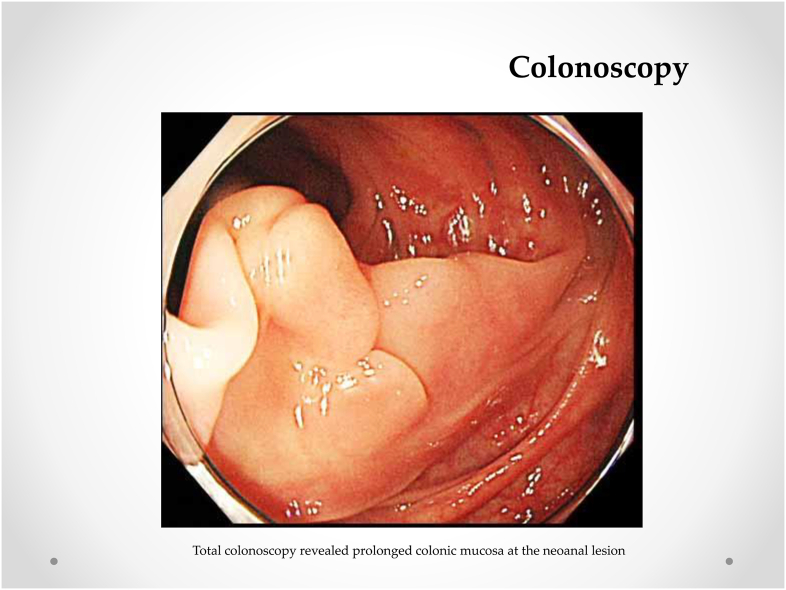
**Colonoscopy.** Total colonoscopy revealed prolonged colonic mucosa at the neoanal lesion.

A 75-year-old Japanese man who was suffering from lower abdominal pain visited our hospital. A total colonoscopy revealed two lesions in the rectum below the peritoneal reflection (3.5 cm and 15 cm above the anal verge). The patient underwent a radical laparoscopic intersphincteric resection through the transanal approach, with diverting ileostomy and lymphadenectomy, to treat double rectal cancer (Fig. 1). The final pathological diagnosis was a moderately differentiated tubular adenocarcinoma invading muscularis propria without lymph node metastasis in the point of both rectal cancers. Four months later, we performed an ileostomy closure surgery. Five months later, he felt discomfort at the neoanal lesion during defecation and developed colonic mucosal prolapse, manifested by anal bulge and fecal incontinence. Moreover, this symptom appeared nine months after the initial operation. A total colonoscopy revealed prolonged colonic mucosa at the neoanal lesion (Fig. 2).

Laboratory findings at the time of admission were within normal limits, including tumor marker levels, and showed no signs of recurrence of rectal cancer. The preoperative Wexner score was 10 points. We scheduled the Gant-Miwa-Thiersch procedure for colonic mucosal prolapse after ISR.

## 5 Surgical procedure: The Gant-Miwa-Thiersch procedure

**Fig. 3:**
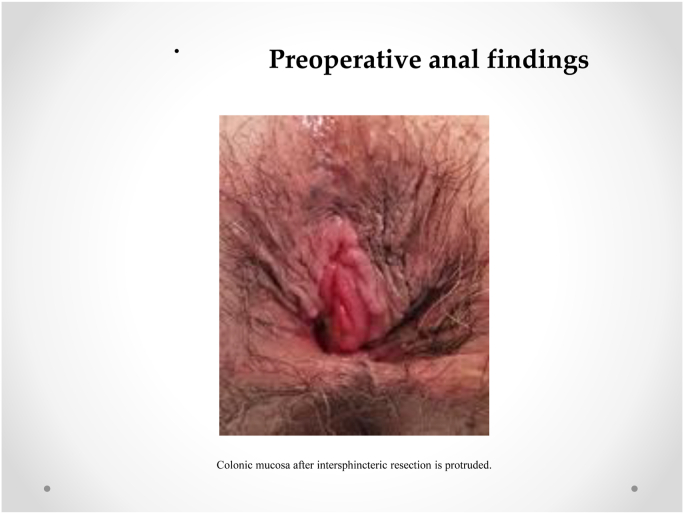
**Preoperative anal findings.** Colonic mucosa after intersphincteric resection is protruded.

**Fig. 4:**
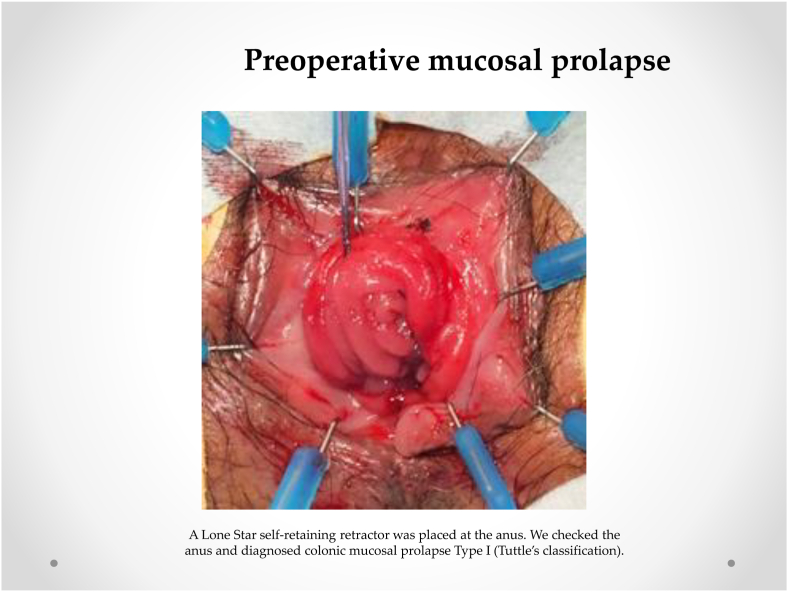
**Preoperative mucosal prolapse.** A Lone Star self-retaining retractor was placed at the anus. We checked the anus and diagnosed colonic mucosal prolapse Type I (Tuttle's classification).

**Fig. 5:**
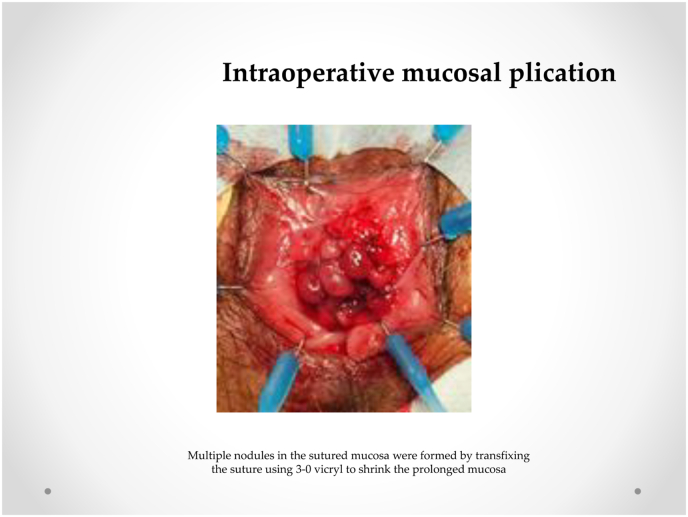
**Intraoperative mucosal plication.** Multiple nodules in the sutured mucosa were formed by transfixing the suture using 3–0 vicryl to shrink the prolonged mucosa.

**Fig. 6:**
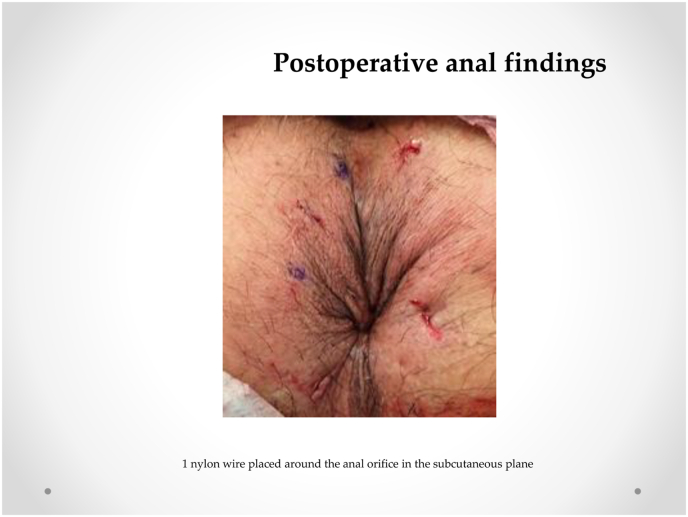
**Postoperative anal findings.** 1 nylon wire placed around the anal orifice in the subcutaneous plane.

Our procedure for prolapsed colonic mucosa is a perineal plication method utilizing nylon wiring. The patient was placed in the lithotomy position under general anesthesia. A Lone Star self-retaining retractor (Lone Star Medical Products, Inc., Houston, TX, USA) was placed at the anus (Fig. 3, Fig. 4). We checked the anus and diagnosed colonic mucosal prolapse Type I (Tuttle's classification: Table 3). Multiple nodules (total 25 times) in the sutured mucosa were formed by transfixing the suture using 3–0 vicryl to shrink the prolonged mucosa (Gant-Miwa procedure) (Fig. 5). We used a 1-nylon wire three times, encircling and straightening the anal canal with a kind of cord above the internal sphincter muscle (Thiersch procedure) (Fig. 6).

**Table 3:**
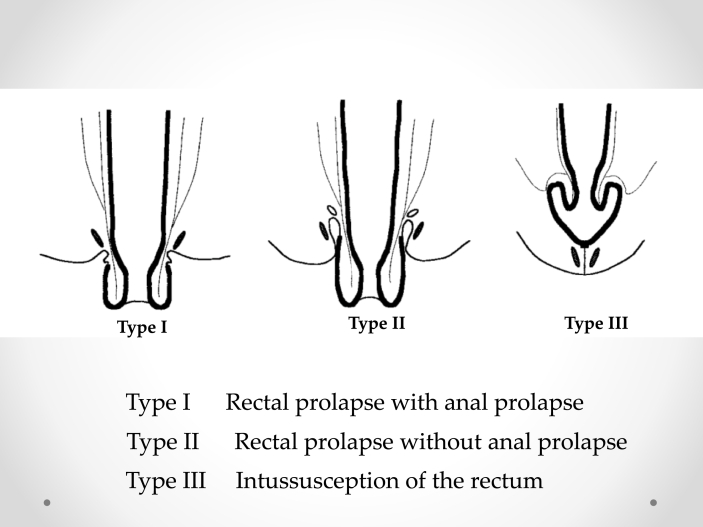
Tuttle's classification for rectal prolapse.

The total operative time was 85 min, and intraoperative blood loss was 5 ml. The patient demonstrated a good postoperative course, and he was discharged from our hospital in remission five days after the operation. The patient's postoperative Wexner score was 4 points. The patient returned for regular follow-up every three months with no mucosal prolapse or rectal cancer recurrence.

## 6 Discussion

Rectal prolapse is regarded as a protrusion of the full thickness of the rectum through the anal canal [9]. Mucosal prolapse should be distinguished from the full-thickness of the rectum if only the rectal or anal mucosa is protruding. In any case, rectal prolapse, including mucosal prolapse, can cause patients to experience various symptoms, such as anal incontinence, constipation, mucus discharge, and hemorrhage, which impede normal bowel function [9,10].

A radical surgical procedure for lower rectal cancer treatment is ISR, which is the ultimate method and is particularly challenging for the patient, as symptoms of anal dysfunction remain unresolved. Even various symptoms related to the anus or neoanus after low anterior resection of the rectum remain unresolved and are severe complications [11].

Factors related to post-ISR dysfunction are preoperative chemoradiation therapy for advanced lower rectal cancer, being male, and extended resection of the anal sphincter muscle [11]. ISR is a recognized risk factor of bowel dysfunction. ISR is a resection of the internal anal sphincter. Sometimes it involves a degree of the internal anal sphincter and a degree of the partial external anal sphincter. Even partial resection of the internal anal sphincter muscle can cause eversion of the anal skin and anal canal mucosa, which is called mucosal prolapse. Colonic mucosal prolapse is an uncommon complication of the ISR.

Worsening mucosal prolapse after ISR worsens anal dysfunction. That is why surgery is the only definitive and curative treatment option for rectal or colonic mucosal prolapse to resolve anal dysfunction.

Although many different surgical procedures have been performed, using either the laparoscopic or open approach to the abdominal procedure uses the same surgical steps among all surgeries, which involve rectal mobilization with fixation of the rectum to the sacrum sutures or by a mesh. According to Tsunoda's review article [9], the results of perineal procedures are rare in the English literature. The Delorme procedure, perineal rectosigmoidectomy (Altemeier procedure), and Gant-Miwa procedure, which is the plication procedure for herniated rectal mucosa followed by narrowing the anal canal using a prothesis (the Thiersch procedure), have been widely used in Japan.

In Japan, simple transanal procedures have been applied for rectal prolapse rather than extensive laparotomy, which is more common in Europe and United States. For these reasons, we investigated the Japanese literature, which included reports on abdominal procedures—13.5% reports on the Sudeck procedure, 2.3% on the Ripstein procedure, 3.3% on abdominal wall surgery—and reports on peritoneal procedures—21% on the Thiersch procedure, 23.8% on the Gant-Miwa procedure, 20.0% on the Delorme procedure, and 3% on the Altemeier procedure [12]. However, following ISR, it is almost impossible to perform an abdominal procedure, so performing a post-ISR peritoneal procedure is better. At our institution, we are continuing to perform the Gant-Miwa-Thiersch procedure as the first-choice treatment for rectal and colonic mucosal prolapse because it is simple and less invasive [13].

The outcome of the Gant-Miwa-Thiersch procedure at our institution was no recurrence and no other severe complications. The operation time was under 65 minutes, and blood loss was less than 7 ml, which is a low amount for an operation. We suppose that our method's relatively optimal clinical outcome was not due to the Gant-Miwa procedure but the incorporation of the Thiersch procedure.

Mucosal plication using the Gant-Mowa or Delorme procedure prevents recurrences. However, mucosal plication can be performed many times. Our Thiersch procedure involves encircling and straightening the anal canal with a 1-nylon wire to fix the anal sphincter. This procedure is not infectious, remains sturdy for a long time, and requires encircling only a few times. It is key to perform this procedure for severe colonic mucosal prolapse after ISR because ISR entails resectioning the inter anal sphincter. In our cases, we resolved mucosal prolapse (Fig. 3) due to the Thiersch procedure (Fig. 6).

Anal encircling combined with the Gant-Miwa procedure or Delorme procedure has proven to reduce the recurrence rate [14]. Certainly, the Delorme procedure after ISR is a good treatment option that provides the clinical benefits of improving local anal symptoms and slightly improving anal function [15,16]. However, the Thiersch procedure, which entails encircling the anal canal three times with nylon, supports repairing the new internal anal sphincter.

In conclusion, the Gant-Miwa-Thiersch procedure is technically simple and can be performed many times [14]. It is a viable treatment option for rectal and colonic mucosal prolapse, especially after intersphincteric resection.

## Funding

This research received no specific grant from any funding agency in the public, commercial, or not-for-profit sectors.

## Source of funding

No specific funding was received for the present analysis.

The authors declare that there are no conflicts of interests.

## Provenance and peer review

Not commissioned, externally peer-reviewed.

## Declaration of competing interest

None.
